# A Case of Brodie’s Abscess With Tibial Erosion and Extravasation Into Surrounding Soft Tissue

**DOI:** 10.7759/cureus.8592

**Published:** 2020-06-13

**Authors:** Josh Lowe, Rachel E Bridwell, Aaron G Matlock, Amber Cibrario, Joshua Oliver

**Affiliations:** 1 Emergency Medicine, San Antonio Military Medical Center, San Antonio, USA; 2 Emergency Medicine, Brooke Army Medical Center, Fort Sam Houston, USA; 3 Emergency Medicine, Brooke Army Medical Center, Fort Sam Houston, San Antonio, USA

**Keywords:** brodie's abscess, pyogenic osteomylitis, atraumatic limp

## Abstract

Atraumatic limb pain and limp is a common pediatric presentation in the emergency department in the United States. In a majority of cases, these presentations are benign. However, in cases where pediatric patients are repeatedly presenting for atraumatic limb pain, further investigation is required. We present such the case of a 14-year-old female with acute worsening of progressive atraumatic knee pain who was found to have a Brodie’s abscess, a subacute pyogenic form of osteomyelitis. This is a particularly challenging diagnosis, as it often presents with no associated symptoms such as fever or weight loss. The consequences of missing this diagnosis include permanent disability and potential amputation, but excellent outcomes can be expected for those who undergo timely surgical debridement and irrigation. We discuss the etiology, common presentations, and treatment of this rare but potentially limb-threatening disease in the hope that clinicians will consider this diagnosis in cases of persistent or progressive atraumatic limb pain.

## Introduction

Atraumatic limb pain and limp is a common pediatric presentation in the emergency department (ED), accounting for 1 out of every 58 pediatric ED visits in the United States [1]. Brodie’s abscess is a rare form of subacute pediatric osteomyelitis that results in a localized bone abscess and presents as an atraumatic limp in pediatric patients. The disorder affects 0.185 per 100,000 children yearly and is twice as common in males [2]. The average age at presentation is 17 years [3]. This diagnosis may be missed multiple times by emergency medicine and primary care physicians, as there are relatively few clinical indicators other than slowly progressive limb pain. We report a case of Brodie’s abscess, which was diagnosed after multiple visits and had eroded into the surrounding soft tissue.

## Case presentation

A 14-year-old female presented to the ED for ongoing right knee pain that acutely worsened over the prior four days. Her medical history was limited to mild intermittent asthma. Over the preceding four months, she was evaluated at a local urgent care, an outside ED, and her primary care physician diagnosed her with pretibial bursitis. She denied trauma to the area and reported that her pain had been constant but tolerable until four days prior, when she developed worsening pain and her shin became warm to touch. She denied any fevers, rashes, or focal neurologic deficits.

On examination, the patient was afebrile with normal vital signs. Clinical examination was notable for mild swelling and warmth overlying the right proximal tibia. The right lower extremity was neurovascularly intact, and the patient could ambulate with a limp. Laboratory evaluation showed a mild leukocytosis of 13,800 cells/uL and an elevated C-reactive protein (CRP) of 11.7 mg/dL. The remaining laboratory results were within normal limits.

**Figure 1:**
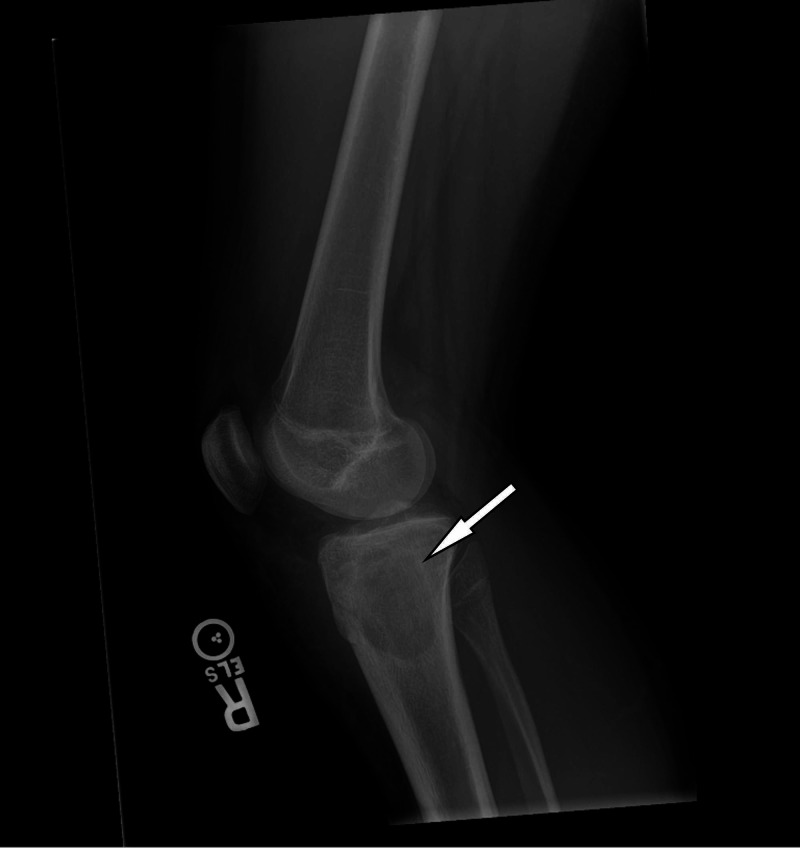
Lateral radiograph, with arrow demonstrating a lucency in the proximal right tibia.

**Figure 2:**
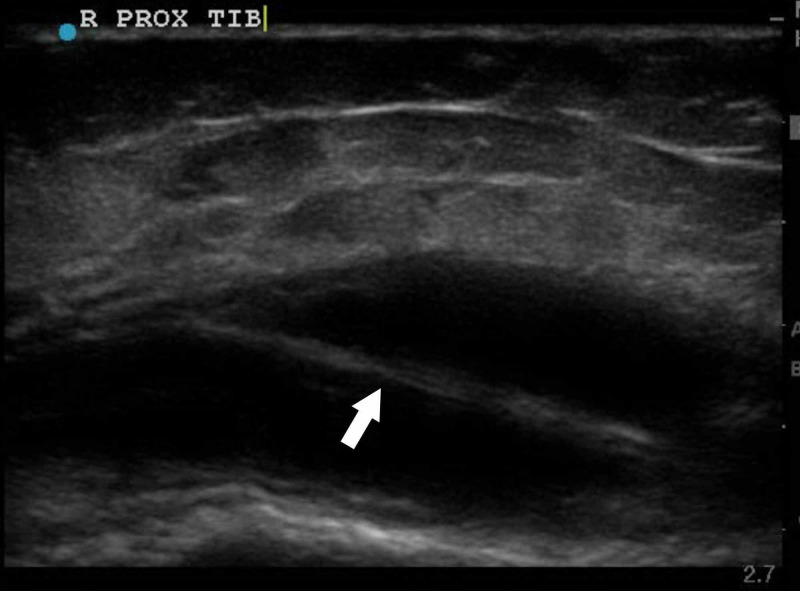
Longitudinal view of the proximal tibia demonstrating fluid collection, with arrow showing large septation (later demonstrated to be fascia) on bedside ultrasound.

**Figure 3:**
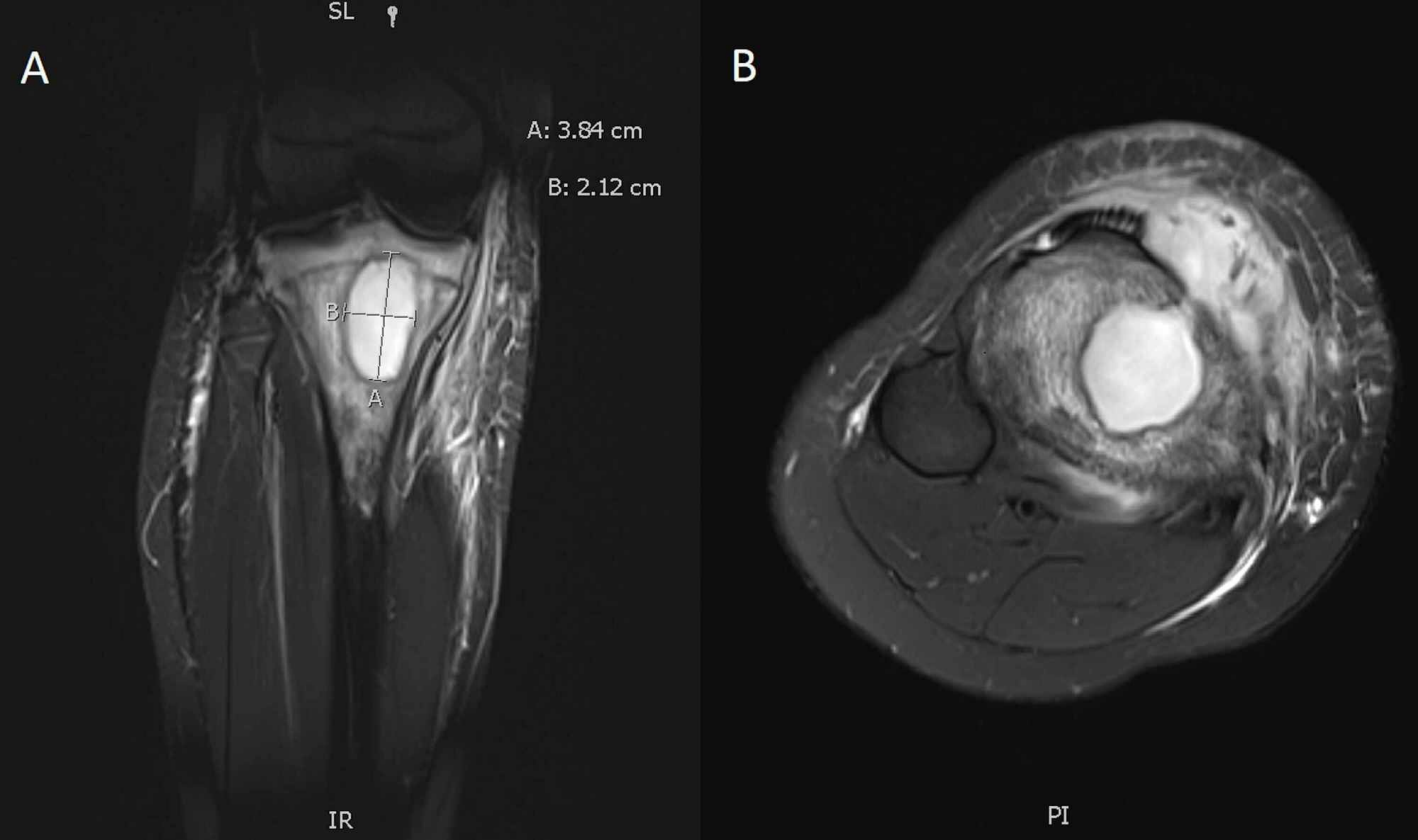
(A) Coronal view of T1 MRI with contrast demonstrating Brodie’s abscess in the right proximal femur. (B) Axial view of T1 MRI with contrast demonstrating Brodie’s abscess in the right proximal femur with a break in the cortex and extravasation into the surrounding tissue.

A knee X-ray showed infrapatellar swelling consistent with bursitis as well as a lucent lesion in the tibial metadiaphysis (Figure 1). A bedside ultrasound showed a fluid collection overlying the right proximal tibia with a large septation (Figure 2). A subsequent contrast-enhanced magnetic resonance imaging (MRI) study demonstrated a well-circumscribed focal area of inflammation within the right proximal tibia with erosion through the anterior tibia into the overlying soft tissue (Figure 3).

The patient was given intravenous (IV) analgesia and IV cefazolin, and was transferred to a pediatric hospital where she underwent surgical debridement and was treated with targeted antibiotics. Intraoperative exploration raised concern for potential future amputation due to the extensive erosion. She was discharged to an acute rehabilitation facility on hospital day 7 and remained non-weight-bearing until follow-up eight weeks later.

## Discussion

First described in a lecture for the Medical and Surgical Society in 1832 by Sir Benjamin Brodie, a Brodie’s abscess is a subacute osteomyelitis with an insidious onset [4-5]. Though the primary site is often not identified, Brodie’s abscess is thought to be a secondary bone infection related to hematogenous spread [6]. Making this diagnosis in the ED is challenging, as many disease processes mimic Brodie’s abscess, clinically and radiographically, including eosinophilic granuloma, chondrosarcoma, osteoid osteoma, Langerhans cell histiocytosis, and tuberculosis [7-11]. As a result, the median time to diagnosis has been reported to be approximately 12 weeks in a review of 70 case reports from 1921-2018 [2]. On presentation, only 21% of patients report recent infection and 45% of patients report preceding trauma. Similarly, 84% of patients were afebrile upon presentation, with normal or mildly elevated serum inflammatory markers [2]. Methicillin-sensitive *Staphylococcus aureus* (MSSA) was the most commonly isolated organism, identified in two-thirds of wound cultures [3]. One-quarter of wound cultures revealed no identifiable bacteria [3]. Though MRI is the diagnostic gold standard, plain films may demonstrate areas of lucency and rule out other diagnoses such as acute fracture [3]. Surgical debridement and MSSA antibiotic coverage are the definitive treatments. Recovery may be prolonged, with an average course of 33 months and a chance of recurrence in one out of six cases [3].

There remains a dearth of definitive outcome data in the available literature. This is likely due to the extensive recovery time and limited data beyond case reports and case series. Although complications are rare, a majority of patients fully recover with timely surgical debridement and irrigation [3].

## Conclusions

Due to its rarity, insidious onset, non-specific presentation, and the absence of systemic symptoms, Brodie’s abscess presents a considerable diagnostic challenge to the emergency medicine provider. It is crucial for emergency clinicians to consider this diagnosis in pediatric patients or young adults who present repeatedly with atraumatic leg pain in order to prevent complications, long-term disability, or amputation.
